# Comparison of the dynamics of Japanese encephalitis virus circulation in sentinel pigs between a rural and a peri-urban setting in Cambodia

**DOI:** 10.1371/journal.pntd.0006644

**Published:** 2018-08-23

**Authors:** Juliette Di Francesco, Rithy Choeung, Borin Peng, Long Pring, Senglong Pang, Raphaël Duboz, Sivuth Ong, San Sorn, Arnaud Tarantola, Didier Fontenille, Veasna Duong, Philippe Dussart, Véronique Chevalier, Julien Cappelle

**Affiliations:** 1 Institut Pasteur du Cambodge, Epidemiology and Public Health Unit, Phnom Penh, Cambodia; 2 University of Calgary, Faculty of Veterinary Medicine, Department of Ecosystem and Public Health, Calgary, Canada; 3 Institut Pasteur du Cambodge, Virology Unit, Phnom Penh, Cambodia; 4 Royal University of Agriculture, Phnom Penh, Cambodia; 5 UMR ASTRE, CIRAD, INRA, Université de Montpellier, Montpellier, France; 6 Ministry of Agriculture, Forestry, and Fisheries, Department of Animal Health and Production, Phnom Penh, Cambodia; 7 Institut Pasteur du Cambodge, Phnom Penh, Cambodia; 8 UMR EpiA, VetAgro Sup, INRA, Marcy l’étoile, France; Department of Virology, Armed Forces Research Institute of Medical Sciences, Bangkok, THAILAND

## Abstract

Japanese encephalitis is mainly considered a rural disease, but there is growing evidence of a peri-urban and urban transmission in several countries, including Cambodia. We, therefore, compared the epidemiologic dynamic of Japanese encephalitis between a rural and a peri-urban setting in Cambodia. We monitored two cohorts of 15 pigs and determined the force of infection–rate at which seronegative pigs become positive–in two study farms located in a peri-urban and rural area, respectively. We also studied the mosquito abundance and diversity in proximity of the pigs, as well as the host densities in both areas. All the pigs seroconverted before the age of 6 months. The force of infection was 0.061 per day (95% confidence interval = 0.034–0.098) in the peri-urban cohort and 0.069 per day (95% confidence interval = 0.047–0.099) in the rural cohort. Several differences in the epidemiologic dynamic of Japanese encephalitis between both study sites were highlighted. The later virus amplification in the rural cohort may be linked to the later waning of maternal antibodies, but also to the higher pig density in direct proximity of the studied pigs, which could have led to a dilution of mosquito bites at the farm level. The force of infection was almost identical in both the peri-urban and the rural farms studied, which shifts the classic epidemiologic cycle of the virus. This study is a first step in improving our understanding of Japanese encephalitis virus ecology in different environments with distinct landscapes, human and animal densities.

## Author summary

The number of Japanese encephalitis cases has decreased substantially over the past decades with the implementation of childhood vaccination programs. Japanese encephalitis virus, however, remains the most important cause of acute viral encephalitis in Eastern and Southern Asia, with an estimated 68,000 cases reported annually worldwide. Our results demonstrate that Japanese encephalitis virus circulates intensely both in a rural and a peri-urban setting in Cambodia, which raises important public health concerns as peri-urban areas are densely populated. These results support the importance of changing vaccination recommendations for travelers and of not focusing national immunization programs against Japanese encephalitis solely on rural areas.

## Introduction

Even though the number of cases has decreased substantially over the past decades with the implementation of childhood vaccination programs in many Asian countries, Japanese Encephalitis virus (JEV) remains the most important cause of acute encephalitis in Eastern and Southern Asia [1,2]. According to the latest World Health Organization estimates, approximately 68,000 cases are notified annually, of which 15,000 die. Most of these cases occur in children under 15 years of age [1]. These figures are, however, probably underestimated [2]. In Cambodia, the annual incidence in this age group was estimated in 2008 at 11.1 cases per 100,000, based on sentinel hospital surveillance data from 2006 to 2008 [3]. Furthermore, the geographic range of JEV keeps expanding and now extends from the South of Russia to Northern Australia including Japan, Eastern China, India and South-East Asia [4].

Japanese encephalitis (JE) is caused by a Flavivirus, transmitted mostly by *Culex* mosquitoes. *Culex tritaeniorhynchus* is the primary vector of JE in rural settings, followed by *Culex vishnui* and *Culex gelidus*, whereas anthropophilic species like *Culex quiquefasciatus* are probably more involved in urban and peri-urban JE transmission [5–7]. Ardeid birds (black-crowned night herons and plumed egrets) are historically thought to be the main reservoir for JEV transmission [8]. Domestic pigs are considered the major amplifying hosts, and humans important dead-end hosts [5,9]. The role of ardeid birds and domestic pigs in the transmission cycle of JEV is generally accepted, but the latter is probably more complex than its current description might suggest and possibly varies depending on the environment (peri-urban, urban or rural) [10]. Moreover, JEV also infects a wide range of vertebrates, which may have a role in its transmission, but the extent of their role is uncertain and they are hence currently considered dead-end hosts [9]. Recent studies, however, have shown that young chicks and ducklings as well as several species of passerine birds experimentally developed a viremia sufficiently high to allow virus transmission to mosquitoes [11,12]. Finally, it was thought until recently that JE transmission occurred exclusively through mosquitoes, but a study demonstrated that JEV could be transmitted from pig to pig in the absence of vectors: there is still much to learn about JE’s complex epidemiology [13].

JE is considered mainly a rural disease. Proximity to rice fields and pig rearing, particularly backyard farming, have been identified as major risk factors of JE in humans, and these factors coexist in many rural areas across Cambodia [14,15]. However, several studies conducted in Thailand and Vietnam have shown that JEV and its vectors can be found in peri-urban areas [16–18]. Two longitudinal studies conducted in Cambodia– 2006–2008 and 2010–2013 periods–found that respectively 15% and 24.4% of the encephalitis cases observed at the Kantha Bopha Hospitals in Cambodia were caused by JEV [3,19]. Additionally, a previous study we conducted in the suburbs of Phnom Penh in 2014 showed that all tested pigs had antibodies against JEV before the age of six months [20].

It is, therefore, essential to assess the importance of JEV’s circulation in peri-urban and urban settings and to study its transmission cycle and the species involved with more accuracy. Peri-urban and urban areas are characterized by very high population densities, particularly in southeast Asia, and the circulation of JEV in these types of settings raises important public health concerns. This is especially true in Cambodia where even though vaccination against JE was added to the routine national childhood immunization schedule in March 2016, the continuity of this program is not yet ensured. Finally, it is crucial to better understand the transmission of JEV in non-rural environments in order to assess JE’s risk of emergence in new regions, particularly in Europe, where vectors, amplifying and reservoir hosts of the virus can be found [21–23].

The objective of this study was to describe and compare the epidemiological dynamic of JE between a rural and a peri-urban setting in Cambodia. To do this we determined: i) whether the force of infection–the rate at which susceptible pigs become infected–was lower in the peri-urban study site than in the rural site; and ii) what were the different factors, including mosquito abundance and diversity as well as host densities and environmental characteristics, that could explain a different circulation of JE in both areas. We monitored two cohorts of 15 pigs, respectively in a peri-urban and rural farm, used light traps to capture mosquitoes in proximity of the pig pens during three consecutive nights each month throughout the entire study, and carried out household surveys to characterize both study areas in terms of host densities.

## Materials and methods

### Ethics statement

All research was carried out under an animal care permit from the National Animal Health and Production Research Institute (NAHPRI, former National Veterinary Research Institute–NaVRI). We observed the animal welfare principles of the World Organization for Animal Health’s (OIE) Terrestrial Animal Health Code, Chapter 7.8 “Use of Animals in research and education” [24]. We sampled the animals every eight to 11 days to limit handling and sampling stress. In the peri-urban farm, where animals were not born onsite, we did not sample the pigs during the first two weeks following their arrival in order to give them time to acclimatize to their new environment.

### Study sites and origin of the pigs

**Fig 1:**
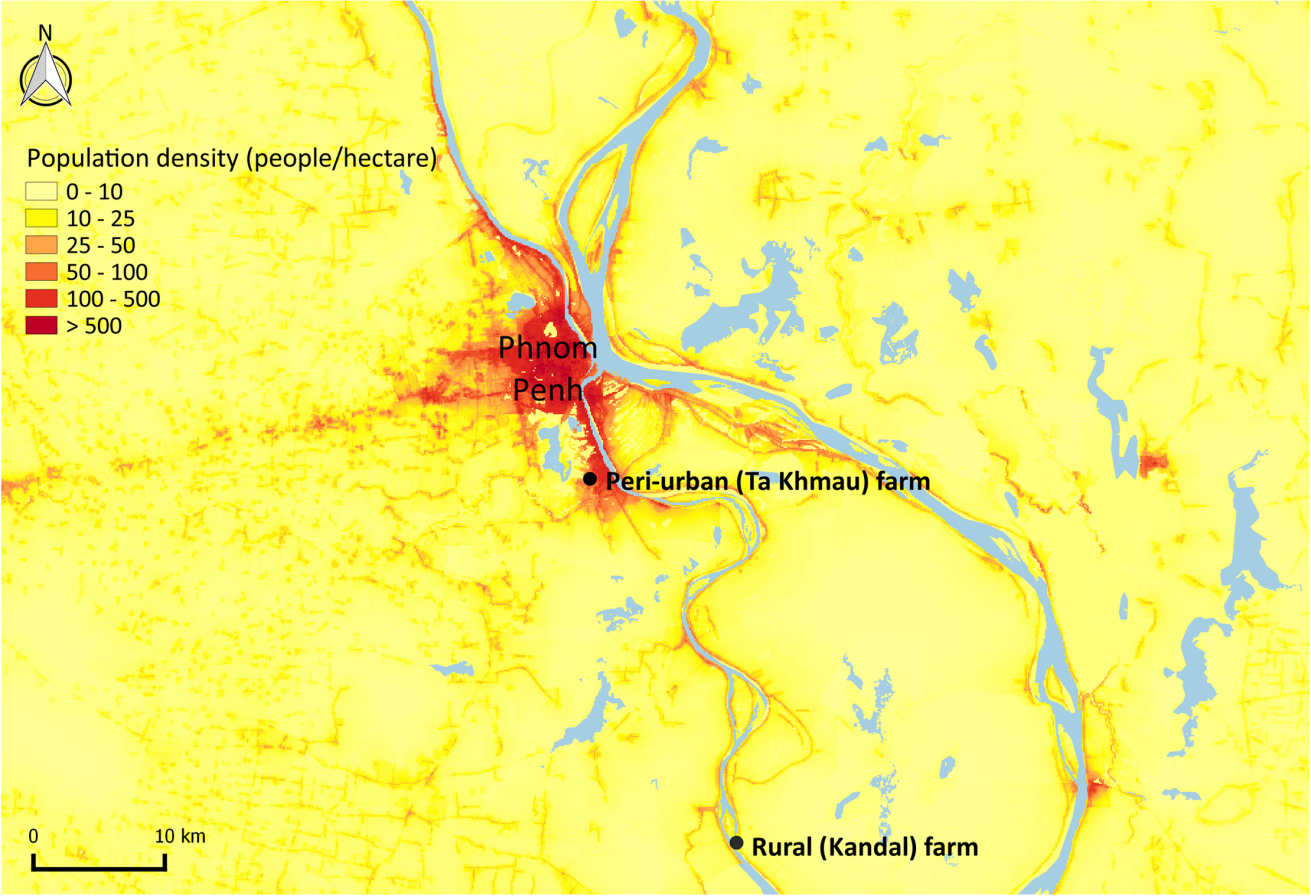
Location of both farms in the peri-urban site (Ta Khmau) and the rural site (Kandal), Cambodia. Data regarding population density are from the year 2015 and were drawn from the WorldPop project [25].

This study was conducted in two farms, located in a peri-urban and a rural area, respectively. Fifteen pigs were raised and followed in each farm. The peri-urban farm was located in Ta Khmau, Kandal province, in the southern suburbs of Phnom Penh, in an area characterized both by a high human density and a mix of urban and rural landscape with cultivated fields (Fig 1).

In the peri-urban cohort, nine of the pigs came from a litter of 13 individuals, born from a five-year-old sow that had already given birth to seven litters. The six remaining pigs came from a litter of 11 individuals, born from a two-year-old sow that had already given birth to two litters. All the pigs were born on farms situated in the Ta Khmau area on the 15^th^ of April 2015.

The rural farm was a 20-sow backyard farm situated in Kandal province, 60 km south of Phnom Penh (Fig 1). The pigs were all born onsite on April 17, 2015. They came from two different litters, respectively comprised of seven and eight pigs. Both sows were gilts of one and a half years of age.

### Pig sampling

Pigs in the peri-urban farm were ear-tagged C01 to C15, and those in the rural farm D01 to D15. Every eight to 11 days, we sampled blood from each pig at the jugular vein using dry tubes with a Vacuette system.

Results from 500 pig sera collected between 2006 and 2007 in eight Cambodian provinces showed that 95% of the pigs older than six months had been infected by JEV [26]. In regions where JE is endemic, most sows are infected before their sexual maturity and transmit maternal antibodies to their piglets at birth. Piglets lose this immunity generally by two to three months of age [20]. Thus, most pigs in Cambodia are infected between two and six months and this is the age-frame we chose for sampling (July to October 2015).

### Pig sample analyses

In pigs, immunoglobulins M (IgM) against JEV generally appear two to three days post-infection, peak at around one week, and persist approximately two to three weeks [27], whereas IgG develop within several weeks [28]. Their viremia usually remains sufficiently high to infect mosquitoes during two to four days [28,29]. In this study, IgG antibodies were detected in the pig sera using an indirect ELISA test adapted from Yang *et al*. and described in details in Duong *et al*. [26,30]. The optical density (OD) was measured for each sample and the mean OD value M of the three negative controls run on each microplate was used to define the cut-off values. A sample was considered positive if its OD was higher than four times M, negative if its OD was lower than three times M, and equivocal if its OD was between three and four times M.

RT-PCR protocols were used to detect JEV RNA in the serum samples collected during the two weeks preceding each pig’s presumed seroconversion date. JEV isolation on cells was attempted on all RT-PCR positive samples and amplicon sequencing was performed. Methods used for RNA extraction, amplification of JEV RNA using conventional and real-time RT-PCR, JEV isolation on cells, amplicon sequencing, and phylogenetic analyses are detailed in Duong *et al*. [31]. Briefly, for each pig serum sample, the QIAamp Viral RNA Mini Kit (QIAGEN, Hilden, Germany) was used according to the manufacturer’s instructions to extract viral RNA. A specific real time RT-PCR assay previously described by Shirato *et al*. was used to detect the JEV RNA [32]. A conventional PCR using JEV-specific sense and anti-sense primers targeting the NS3 gene was then performed on all real time RT-PCR positive samples, as described previously by Tanaka [33]. A mosquito cell line (clone C6/36 of *Aedes Albopictus* cells) was used to isolate JEV from real time RT-PCR positive samples. Following the manufacturer’s instructions, total RNA was extracted from the original samples or supernatant previously detected positive for JEV using the QIAamp Viral RNA Mini Kit (QIAGEN, Hilden, Germany). cDNA was synthetized and then amplified by PCR for full genome, complete envelope or partial NS3 sequencing by Sanger method at a commercial facility (Macrogen, Seoul, South Korea) [31]. CLC Main Workbench 5.5 package (CLC bio A/S, Aarhus, Denmark) was used to analyze and assemble the sequences generated from the PCR products. JEV sequences were aligned with JEV reference strains available in GenBank and belonging to genotypes I to V of JEV. Finally, phylogenetic analyses were carried out using MEGA 5.2 with 1000 bootstrap re-sampling [34].

### Mosquitoes

One home-made CDC light-trap was placed next to the pig pens during three consecutive nights every month during the entire duration of the study, from July to October 2015. Mosquitoes captured were counted and species were identified using Southeast Asia morphological identification keys [35–37].

### Interviews

Household surveys were conducted within a one-km radius around each farm using standardized questionnaires designed for this study (S1 File) to estimate the human densities and that of different domestic animals. A circle of one km in radius was drawn on Google Earth around each farm and divided into four equal areas. To approximate the demographic weight of each of these four quadrants, we counted the number of houses and subsequently respected the proportion of households they represented in the study area when we defined the number of households to interview per quadrant. We walked along the roads identified using Google Earth and selected households to be interviewed based on residents’ presence at the time of our visit, until we reached the desired number of interviews for each quadrant. Households surveyed were, therefore, not selected randomly, but were to the best of our ability distributed homogeneously across each study area. We calculated human densities using the results from the questionnaires, the surface of the delimited area and the number of houses originally counted. We then estimated domestic animal densities using the ratios of each animal species per inhabitant and the computed human densities.

A Chi-square test was used to compare the proportions of households owning pets and production animals between both study areas with the risk of error α set at 5%, and using R software version 3.1.3 [38].

### Estimation of the force of infection of JEV among the pigs on each farm

We used the same method as Cappelle *et al*. to estimate the force of infection in this study [20]. The force of infection is the rate at which sero-negative pigs become positive, also defined as the instantaneous probability of a susceptible pig to become infectious. It can be expressed by:

$$\frac{dS(t)}{dt}=-\lambda S(t)$$ (1)

Where S(t) is the number of susceptible individuals at time t and λ represents the force of infection.

Eq (1) has for solution:

$${S(t)=S(0)e}^{-\lambda t}$$ (2)

Where S(0) is the number of susceptible individuals at time 0.

Eq (2) can be linearized as:

ln(S(t))=ln(S(0))−λt. (3)

Since ELISA-negative pigs were a baseline indication of the number of susceptible individuals, we had data on the evolution in time of the number of susceptible pigs. We estimated the force of infection using Eq (3) by fitting a generalized linear model with a Poisson distribution for count data. For each cohort, time 0 was defined as the date at which the number of susceptible pigs was the highest and subsequently followed an exponential decrease. We considered that pigs both seronegative and PCR negative were susceptible. We used a time step of one day and considered all ELISA-equivocal and all PCR positive pigs as non-susceptible. All analyses were done using R software version 3.1.3 [38].

## Results

### Pig monitoring

All 29 pigs that remained at the end of the study seroconverted by the age of six months. Serology and virology results of the peri-urban (Ta Khmau) and rural (Kandal) cohorts are detailed in Tables 1 and 2, respectively, as well as in the S1 and S2 Figs. Since immunity against JE is long-lasting in pigs, sampling was stopped before the age of six months in Ta Khmau (Table 1) because all the pigs had two consecutive positive samples. Pigs were, however, kept on the farm until the end of October so that mosquito captures could be done in a similar environment.

**Table 1:** Serological and virological results in the peri-urban cohort (Ta Khmau), June-September 2015, Cambodia. Green cells correspond to ELISA-negative samples, yellow cells to ELISA-equivocal samples, and red cells to ELISA-positive samples.

		Date of Sampling							
		19/06	29/06	10/07	20/07	30/07	10/08	20/08	01/09
Age of pigs (days)		65	75	86	96	106	117	127	137
**Pig Identification Number**	**C05**	**PCR-**							
	**C01**	**PCR-**							
	**C02**	**PCR-**							
	**C03**	**PCR-**							
	**C12**	**PCR+**	**PCR-**						
	**C11**		**PCR-**	**PCR-**					
	**C13**		**PCR-**	**PCR-**					
	**C14**		**PCR-**	**PCR+**					
	**C10**			**PCR-**	**PCR-**				
	**C15**	NA			**PCR-**	**PCR-**			
	**C04**				**PCR-**	**PCR-**	**PCR+**		
	**C08**				**PCR-**	**PCR-**	**PCR-**		
	**C06**					**PCR-**	**PCR-**		
	**C07**					**PCR-**	**PCR-**		
	**C09**					**PCR-**	**PCR-**		

**Table 2:** Serological and virological results, rural cohort (Kandal), June-October 2015, Cambodia. Green cells correspond to ELISA-negative samples, yellow cells to ELISA-equivocal samples, and red cells to ELISA-positive samples.

		Date of Sampling													
		22/06	01/07	13/07	23/07	31/07	11/08	23/08	31/08	09/09	17/09	28/09	08/10	19/10	28/10
Age of pigs (days)		66	75	87	97	105	116	128	136	145	153	164	174	185	194
**Pig Identification Number**	**D06**	**PCR-**													
	**D02**					**PCR-**	**PCR-**								
	**D07**							**PCR-**	**PCR-**						
	**D13**							**PCR-**	**PCR-**	**PCR-**					
	**D08**								**PCR-**	**PCR+**					
	**D03**								**PCR-**	**PCR+**					
	**D05**								**PCR-**	**PCR-**					
	**D01**								**PCR-**	**PCR-**					
	**D12**								**PCR-**	**PCR-**					
	**D09**									**PCR-**	**PCR-**				
	**D10**									**PCR-**	**PCR-**				
	**D11**								**PCR-**	**PCR-**	**PCR-**				
	**D15**											**PCR-**	**PCR+**		
	**D04**											**PCR-**	**PCR-**	**PCR-**	
	**D14**										NA	NA	NA	NA	NA

In the peri-urban farm (Ta Khmau), most pigs remained susceptible only a short time before becoming infected; six pigs were infected within 31 days after losing their maternal antibodies, and one (C06) was infected in less than 41 days. This duration, however, could not be calculated for eight of the pigs as they were either susceptible or already infected at the beginning of the study. The average age at infection was 98 days (C05 and C09 were not included in the calculation as their infection date could not be determined). In the rural farm (Kandal), by contrast (Table 2), only four pigs remained susceptible during less than 30 days (D06, whose exact duration could not be determined since it had already lost its maternal antibodies when we began sampling, D02, D07, and D13). The ten other pigs remained susceptible between 40 and 80 days before becoming infected. All the pigs had lost their maternal antibodies at the age of 116 days. The infection of pig D02 was followed by the infection of all the other susceptible pigs within 60 days and the average age at infection was 150 days.

Pigs’ maternal antibodies waned much later in the rural farm than in the peri-urban farm. Over half the pigs had already lost their maternal antibodies by the age of two months in Ta Khmau whereas they had waned in only three pigs in Kandal at that same age. Moreover, only two pigs had not lost their maternal antibodies by the age of 85 days in Ta Khmau compared with ten pigs in Kandal.

JEV RNA was detected by real time RT-PCR in six of the 29 pigs (C04, C12, and C14 in the peri-urban farm, and D03, D08, and D15 in the rural farm) (Tables 1 and 2). Detailed PCR and sequencing results from Duong *et al*. can be found in the S1 Table. Briefly, full JEV genome sequences were generated from two pigs (C14 and D03), partial sequences of the NS3 gene were generated from four pigs (C04, C12, D08, and D15), and one complete coding sequence of the E (envelope) gene was generated from one pig (D08). All JEV strains from 2015 detected in both the peri-urban and rural farms belonged to genotype I clade b [31].

### Mosquitoes

**Fig 2:**
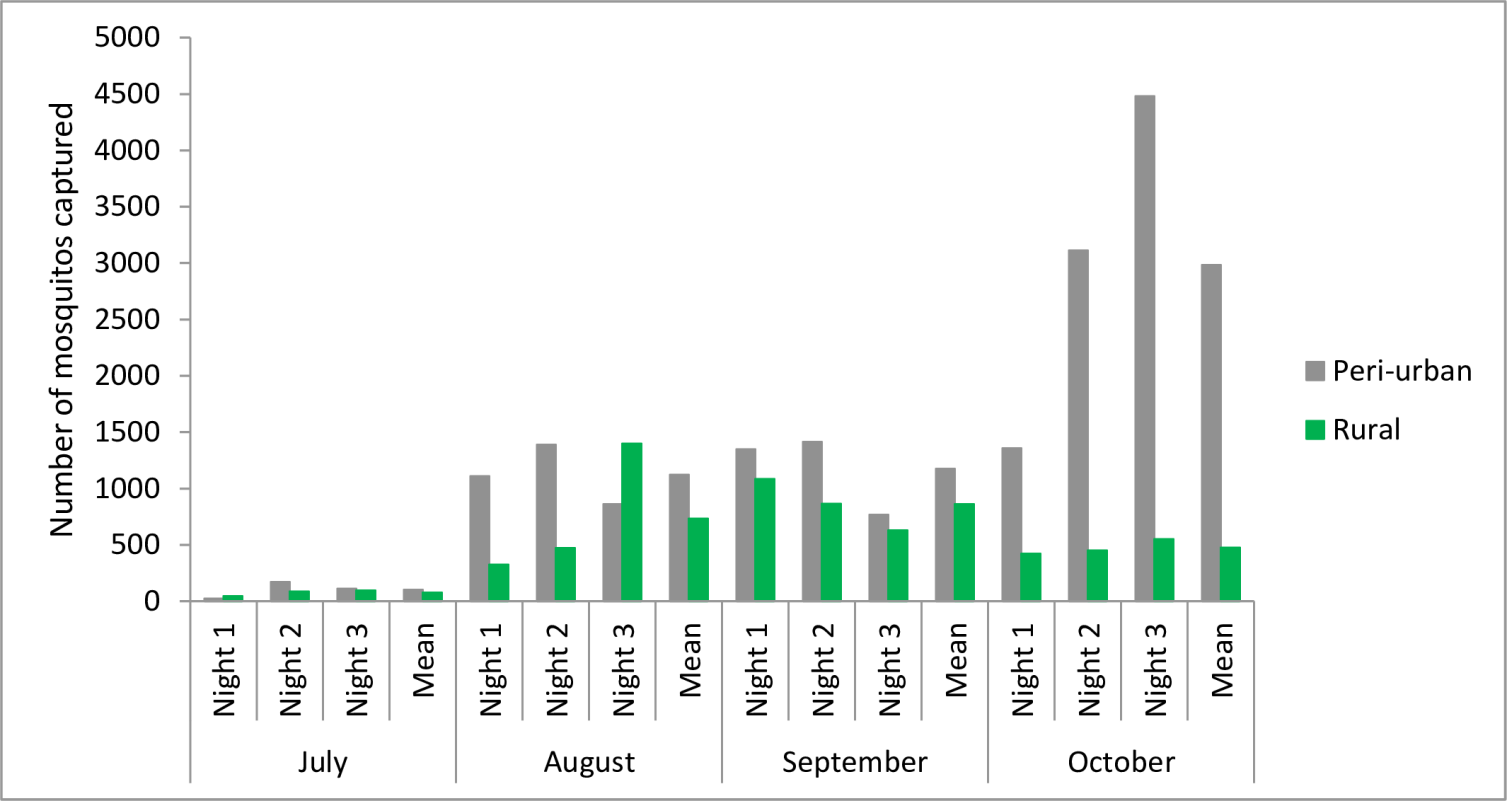
Number of mosquitoes captured per night in Kandal, rural site, and in Ta Khmau, peri-urban site, Cambodia, July-October 2015.

Detailed counts of mosquitoes per species, farm, and month can be found in the S2A, S2B, S2C and S2D Table. The number of mosquitoes captured during two successive nights varied greatly in both sites (Fig 2). Slightly more mosquitoes were consistently captured in the peri-urban farm (Ta Khmau) between July and September, and this difference was greater than six-fold in October. In Ta Khmau, the number of mosquitoes captured increased from July to October, whereas in Kandal, it increased from July to September, but decreased in October.

**Fig 3:**
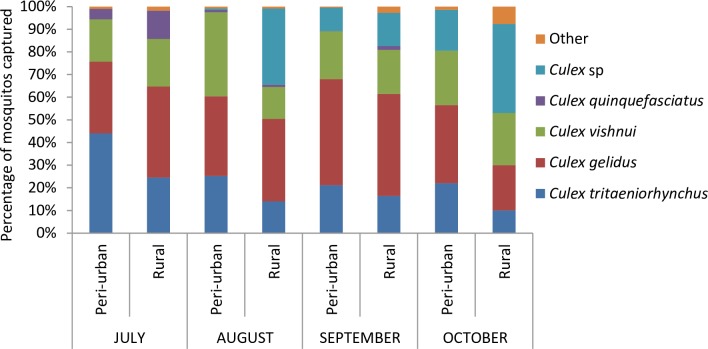
Percentages of the mosquito species captured, females only, July-October 2015, Ta Khmau, peri-urban site, and Kandal, rural site, Cambodia. Species classified in the category “Other” include *Anopheles* sp, *Aedes albopictus*, *Mansonia* sp, *Armigerus* sp, etc.

The proportions of the different species captured are presented for female mosquitoes in Fig 3. The three most abundant species were *Culex* (*Cx*.) *tritaeniorhynchus*, *Cx*. *gelidus*, and *Cx*. *vishnui*. Very few *Cx*. *quinquefasciatus* were captured in both farms (usually between 0 and 1% depending on the month except for July).

### Interviews

Approximately 2,365 households were counted within one km around the peri-urban farm and 530 were similarly counted around the rural farm. We interviewed 280 (11.8%) and 138 (26.0%) households in Ta Khmau and Kandal, respectively. The mean number of persons per household was estimated using the results from the interviews, and was 5.8 (SD = 2.6) in Ta Khmau and 5.1 (SD = 2.2) in Kandal. The population density was then calculated in both areas, at 4,363 inhabitants/km^2^ in the peri-urban site and at 857 inhabitants/km^2^ in the rural site.

The ratio of pigs per inhabitant was 3.96 times greater in the rural site, that of cattle per inhabitant 11.36 times greater and that of chickens twice higher (Table 3). The ratios of dogs, cats, and ducks per inhabitant differed only slightly between both study areas (Table 3). The estimated density of cattle was 2.26 times greater in the rural area, whereas the densities of the other domestic species were between 1.3 and 8 times greater in the peri-urban area (Table 3).

**Table 3:** Comparison between both study areas of the ratios per inhabitant and the densities of the different domestic animal species raised, Ta Khmau and Kandal, Cambodia, 2015.

	Dogs	Cats	Cattle	Pigs	Ducks	Chickens
Animal/pers ratio rural	0.19	0.039	0.23	0.17	0.090	1.39
Animal/pers ratio peri-urban	0.15	0.062	0.020	0.044	0.072	0.69
**Animal/pers ratio rural/peri-urban**	**1.27**	**0.62**	**11.36**	**3.96**	**1.26**	**2.03**
Density rural (animals/km^2^)	162.83	33.42	197.11	145.69	77.13	1,191.23
Density peri-urban (animals/km^2^)	654.45	270.51	87.26	191.97	314.14	3,010.47
**Density ratio rural/peri-urban**	**0.25**	**0.12**	**2.26**	**0.76**	**0.25**	**0.40**

The proportions of households with pets (dogs and/or cats) were 45.7% and 53.6% in Ta Khmau and Kandal, respectively, and they did not differ between both study areas (Chi^2^ = 2.32; p = 0.128). The proportion of households raising production animals (cattle, pigs, ducks, and/or chickens) was significantly higher in the rural site (29.3% in Ta Khmau versus 77.5% in Kandal; Chi^2^ = 86.88; p < 0.001).

### Estimation of the force of infection of JEV in the sentinel pigs

**Fig 4:**
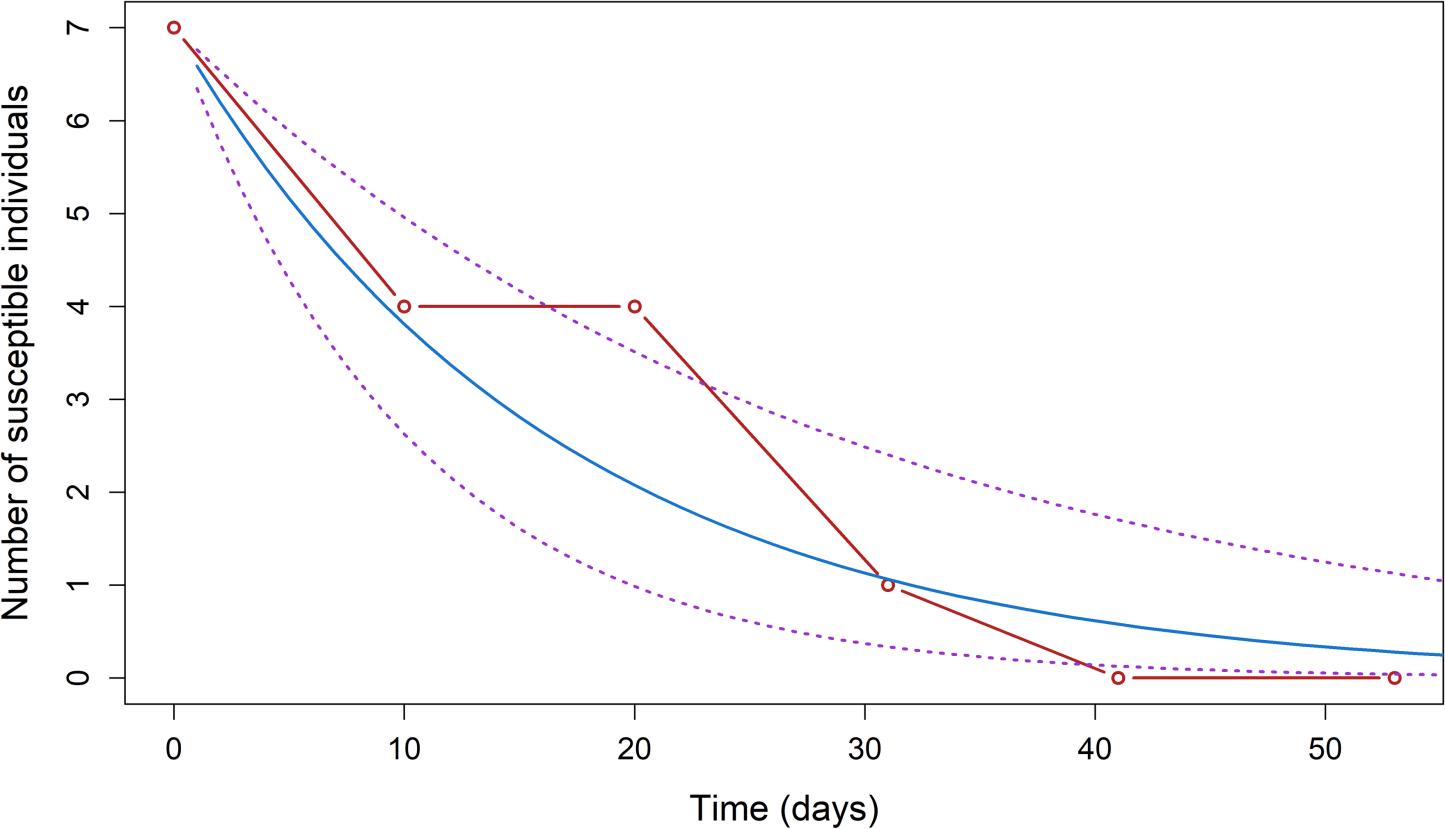
Progression of the number of susceptible pigs S(t) observed (red) and predicted (blue) using a generalized linear model with the 95% confidence interval (dotted purple lines) for the Ta Khmau peri-urban cohort (λ = 0.061 (0.034–0.098); S(0) = 7), Cambodia, 2015. Time 0 on the graph was defined as the date at which the number of susceptible pigs was the highest and subsequently followed an exponential decrease.

**Fig 5:**
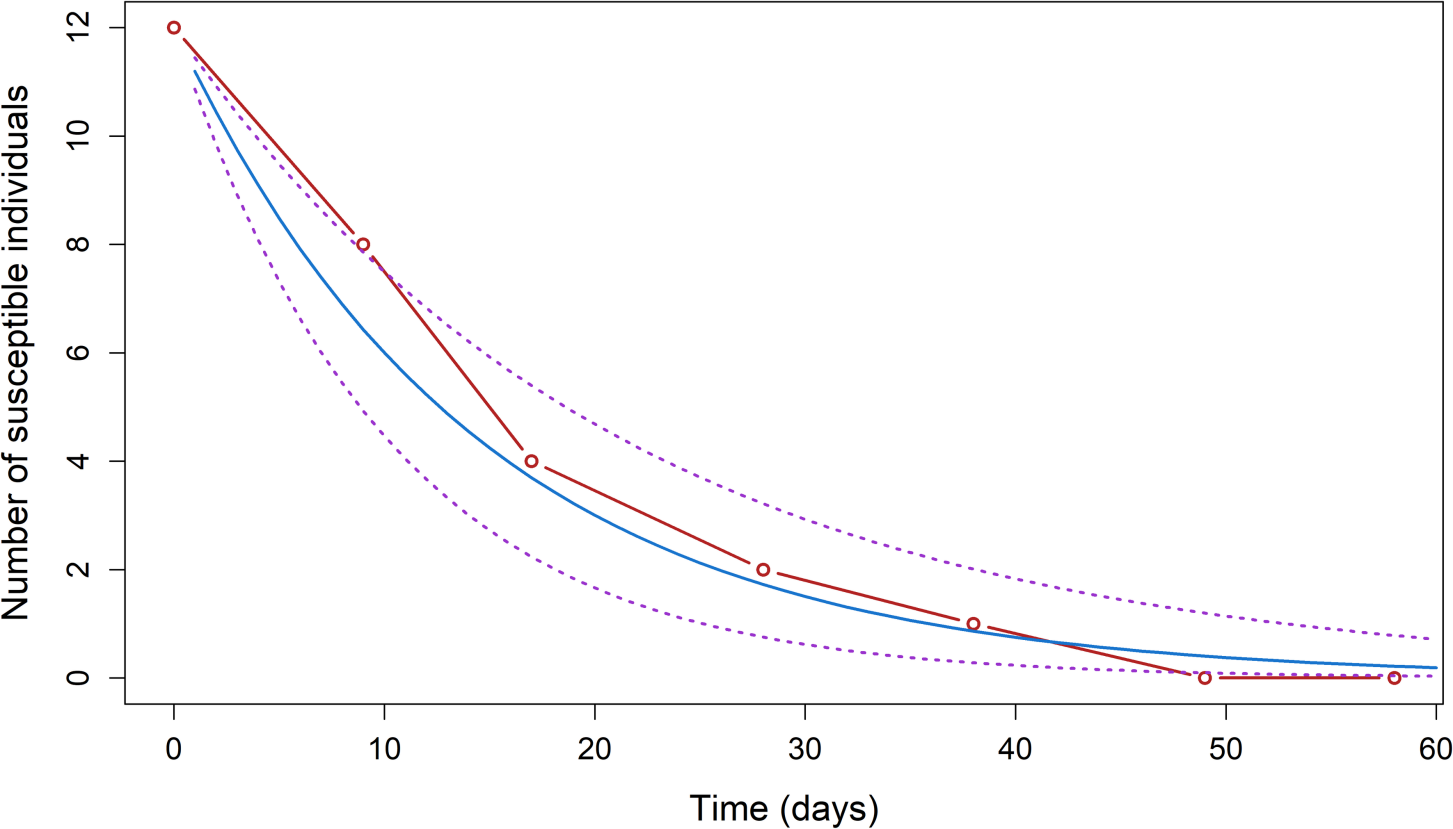
Progression of the number of susceptible pigs S(t) observed (red) and predicted (blue) using a generalized linear model with the 95% confidence interval (dotted purple lines) for the Kandal rural cohort (λ = 0.069 (0.047–0.099); S(0) = 12), Cambodia, 2015. Time 0 on the graph was defined as the date at which the number of susceptible pigs was the highest and subsequently followed an exponential decrease.

July 10^th^ 2015 (date of the third blood sample) was the starting date for the calculation of the force of infection in the peri-urban cohort, with seven susceptible pigs. The estimated force of infection was 0.061 per day (95% confidence interval (CI 95%) = 0.034–0.098); during that period, a susceptible pig had a 6.1% probability of getting infected by JEV every day (Fig 4). August 31^rst^ 2015 (date of the 8^th^ blood sample) was the starting date for the calculation of the force of infection in the rural cohort, with 12 susceptible pigs. The estimated force of infection was 0.069 per day (CI 95% = 0.047–0.099): during that period, a susceptible pig had a probability of 6.9% of being infected by JEV every day (Fig 5).

## Discussion

All pigs in both study farms seroconverted before the age of six months. These results confirm those of Duong *et al*. and those of Cappelle *et al*. showing that JEV circulates intensely in rural, but also in peri-urban settings in Cambodia [20,26]. They also complement the studies carried out by Lindahl *et al*., which evidenced the circulation of JEV in an urban environment in Southern Vietnam for the first time [17,18].

Despite its growing importance in urban settings, JE remains a mainly rural disease [9,15]. We consequently expected to document a higher force of infection in the rural farm, but calculated similar forces of infection (0.061 per day (CI 95% = 0.034–0.098) in the peri-urban farm and 0.069 per day (CI 95% = 0.047–0.099) in the rural farm). However, these forces of infection were calculated at only one rural and one peri-urban site and prevent from any generalization of the results. Additional studies in similar environments would be necessary to confirm that these results are not dependent on specific conditions at our study sites, including farming practices or local environment.

The force of infection measured in the peri-urban farm is consistent with previous results. In 2014, two cohorts of 15 pigs were successively studied in the same farm [20]. The forces of infection determined for the cohorts monitored from April to July 2014 and from September 2014 to January 2015 were 0.032 per day (sd = 0.0056 per day) and 0.046 per day (sd = 0.008 per day), respectively [20]. In this study, we estimated a force of infection of 0.061 per day (CI 95% = 0.034–0.098), which is higher than the ones previously documented but within the same range. We began sampling pigs in July during this study, at the peak of the rainy season, instead of its onset (May) or end (September) in 2014. The slight differences may reflect seasonal variations of the force of JEV infection; additional data collected year-round would be necessary to assess such seasonal trends.

We were able to detect six JEV strains by PCR from the pigs during this study. With a viremia of two to four days, a probable PCR detectability of JEV viremia of approximately two days and samples collected every 10 days, we expected to detect the virus by real time RT-PCR in approximately 1/5 of the pigs [28,29]. This corresponds to our detection rate of 6/29. By contrast, during the previous study, only two out of the twenty-nine pigs monitored tested positive by real time RT-PCR [31]. Since the same detection methods were used in 2014 and 2015, the difference in the detection rates is difficult to explain and the lower detection rate in 2014 might only be due to chance. We were able to detect JEV viremia in a total of eight pigs over the two years, demonstrating the usefulness of our protocol to monitor the dynamics of this virus that is particularly difficult to detect by RT-PCR and then to isolate on cells [31]. As highlighted by Duong *et al*., the same JEV strains (belonging to genotype I clade b) circulated in the peri-urban and rural farms in 2015, and differed from the strain circulating the previous year in the peri-urban farm [31].

All the pigs became infected very rapidly in both farms after the infection of the first individual. However, while all of them seroconverted very rapidly in the peri-urban farm after the waning of maternal antibodies, most pigs remained susceptible during several weeks in the rural farm before becoming infected. This difference in the delay before the first infection may have several explanations.

First, there was an important difference in the pig abundancies between the two farms in which the cohorts were reared. The peri-urban cohort was reared in an experimental farm, used exclusively for research purposes to rear the 15 pigs of the study, whereas the rural cohort was reared in a backyard farm among 120 other pigs of different ages. The pig density in direct proximity with our pigs was, therefore, much higher in the rural farm, which may have led to a dilution phenomenon of mosquito bites and delayed the infection of the first pigs.

Second, in addition to this farm level mosquito bite dilution, a possible area effect may explain the difference observed between the two sites. The number of mosquitoes captured was always slightly higher in the peri-urban farm (Ta Khmau), except in October, where it was substantially higher (Fig 2). This difference in October, however, did not contribute to the differences observed, as almost all the pigs were already infected. It is important to note that our capture methodology enabled us to measure only the relative abundance of mosquitoes, rather than their absolute abundance, which would have required also studying larval breeding sites. If the absolute abundance of mosquitoes in both study areas was comparable, a site-level dilution phenomenon may also have contributed to our results. Indeed, the mosquito species captured in abundance in both areas were mainly *Cx*. *triataeniorhynchus*, *Cx*. *gelidus*, and species of the *Cx*. *vishnui* group (Fig 3). All these are known to have a high trophic preference for cattle [39–41], and even though it was very roughly estimated, we found a higher density of these preferred hosts in the rural area. This may have led to a lower mosquito bite rate per pig, and may consequently contribute to explaining the longer duration before the first pig became infected. It is, however, important to note that pig D06 became infected at the very beginning of the study in the rural farm, but that its infection was not followed by local virus amplification, probably because most pigs were still protected by maternal antibodies.

Finally, our results could be linked to a higher number of mosquitoes infected by JEV in the peri-urban site (Ta Khmau), but such a difference could not be highlighted as mosquitoes were not screened for JEV. Results from 2014 showed a minimum infection rate (MIR) of 0.091/ 1,000 for females from all species during the entire study in Ta Khmau [24]. Because of this low MIR, we decided not to screen mosquito pools for JEV using RT-PCR in this study.

Despite the consistency of our results with a previous study at the same peri-urban site, it is important to note that the similar forces of infection measured are not generalizable to all peri-urban and rural settings in Cambodia as we studied only two farms. Moreover, the absence of difference that we found may also be due to the low number of animals included in this study, as evidenced by the large confidence intervals obtained. Additionally, although the sensitivity and specificity of the ELISA test we used have not been fully evaluated, which may represent another limit of this study, this test has been previously correlated with hemmaglutination inhibition and plaque reduction neutralization test results [25, S3 Table].

There were no rice fields near the peri-urban farm, where JEV circulated intensely. An important JEV transmission risk factor is the proximity to irrigated agricultural fields, and particularly to rice paddies [9]. These settings are ideal for JEV transmission to occur [9,15]. The preferred habitat of the main JEV vectors (*Cx*. *tritaeniorhynchus*, *Cx*. *Vishnui*, and *Cx*. *gelidus*) are rice paddies [5,6,15], and wild aquatic birds generally feed in this type of environment. Although the reservoir role of Ardeid birds in the epidemiologic cycle of JE is considered established, it is important to note that very few studies have clearly proved this, and even less in Cambodia [8,42,43]. The “JEV reservoir = paddy field aquatic birds” dogma doesn’t seem to hold here, and echoes the paper of Lord *et al*. that prompts us to rethink the transmission cycle of JE, depending on the context and species present [10]. Our results could suggest, for example, that other wild bird species such as passerines may also play a role as reservoir of JEV. These are a known reservoir of West Nile virus [44,45], another flavivirus phylogenetically and epidemiologically closely related to JEV [46], and experimentally develop a sufficient viremia to allow JEV transmission [12]. Furthermore, certain domestic animal species, such as chickens and ducks, may also be involved in the amplification cycle of JEV both in peri-urban and rural settings. Recent studies indeed found that young chicks and ducklings experimentally infected by JEV developed a sufficient viremia to infect *Culex* mosquitoes, and that the intensity of this viremia diminished with age at the time of infection [11,47].

Maternal antibodies waned much later in the rural farm (Kandal). Because of placental impermeability, pigs are born without maternal antibodies, and acquire this immunity passively through colostrum after birth [48]. Pigs in Kandal probably had more antibodies transferred by their mothers, either because the sows had higher antibody titers against JEV, or because colostrum transfer was better. In Kandal, the sows that gave birth to our pigs were younger and presumably had been infected by JEV more recently, which may have led to higher antibody titers. The pigs in Kandal also were from smaller litters (seven and eight pig litters in Kandal versus 11- and 13-pig litters in Ta Khmau) and perhaps received more colostrum after birth. Finally, there is probably an individual variability in intestinal permeability and absorption efficiency of maternal antibodies [48]. The later waning of maternal antibodies in Kandal is a limit of this study, as pigs in both cohorts did not become susceptible at the same time and the force of infection could not be calculated over the same period.

The number of mosquitoes captured was low in July, moderate in August and September, and high in October. These results are linked to differences in mosquito population dynamics, themselves highly related to temperature and rainfall [9]. Several studies have shown that the occurrence of JE is associated with temperature and rainfall during the two preceding months [49,50]. Rain would indeed trigger an increase in the number of mosquitoes and the temperature would facilitate their multiplication, increase JEV infection rates, and decrease the extrinsic incubation period [9,50]. Other studies have also shown an increase in mosquito populations following high rainfall in Sri Lanka [51,52]. In Cambodia, the rainy season occurs between May and October, but specific rainfall intensities, and the impoundment of mosquito breeding sites, probably contributed to the differences observed between months and study areas.

As expected, *Cx*. *triateanyorhynchus*, *Cx*. *gelidus*, and members of the *Cx*. *vishnui* group were all abundant in proximity to the pigs. Surprisingly, very few *Cx*. *quinquefasciatus* were captured in the peri-urban farm, although they are known to be urban mosquitoes. This could be linked to their high trophic preference for humans [39–41], which would have limited their attraction to the traps placed in proximity to pigs. Breeding sites of the species *Cx*. *tritaeniorhynchus*, *Cx*. *vishnui*, and *Cx*. *gelidus* are flooded fields, whether it is by rain or irrigation (nitrogen-rich waters: ponds and rice paddies, notably following their impoundment) [5,6,15]. Yet, this type of breeding site is usually found in rural areas. However, the peri-urban site was at the interface between an urban and a cropping area (fields other than rice paddies), which explains the simultaneous presence of a high human density and high numbers of rural mosquitoes.

### Conclusion

Our results show that JEV circulates intensely both in a peri-urban and a rural setting in Cambodia, with a force of infection almost identical in both the peri-urban and the rural farms studied. This absence of difference shifts the classic epidemiologic cycle of the virus and this study is a first step in improving our understanding of JEV ecology in different environments with distinct landscapes, human, and animal densities. This knowledge can be important for the implementation of vaccination campaigns and guidance for travelers. For example, vaccination against JE is currently only recommended for travelers to Cambodia who are staying in urban areas for a long period of time or in rural areas for short stays. However, our results show that travelers visiting urban areas at all should also be advised to be vaccinated against JE. Measuring parameters such as the force of infection is also very important to calibrate mathematical models that can be used to better understand the complexity of vector-borne diseases in general and to theoretically test the impact of different control plans and their possible combinations. This is particularly important in countries like Cambodia, where the continuity of the JE vaccination program is not yet ensured. Finally, the protocol we developed to monitor JEV, a virus that is particularly difficult to isolate, was very successful in detecting JEV among pigs. It could be easily applied in other areas of Cambodia and neighboring countries to improve our knowledge of circulating JEV strains both in rural and urban settings.

## Supporting information

S1 File Questionnaire used for the household surveys. (PDF)

S1 Fig Average optic density measured depending on the type of IgG antibody kinetic observed, Ta Khmau cohort, 2015. The optic density of each sample was divided by 3 times the mean absorbance of the three negative controls in the corresponding ELISA plate and then their average was calculated for each sampling date and type of IgG kinetic. The blue line corresponds to the negativity threshold, the red curve to the pigs with an increasing IgG antibody kinetic (C01, C02, C03, C05, and C12), and the green curve to the pigs with a U-shaped IgG antibody kinetic (all other pigs).
(PDF)

S2 Fig Average optic density measured depending on the type of IgG antibody kinetic observed, Kandal cohort, 2015. The optic density of each sample was divided by 3 times the mean absorbance of the three negative controls in the corresponding ELISA plate and then their average was calculated for each sampling date and type of IgG kinetic. The blue line corresponds to the negativity threshold, the red curve to the pigs with an increasing IgG antibody kinetic (D06 only), and the green curve to the pigs with a U-shaped IgG antibody kinetic (all other pigs).
(PDF)

S1 Table PCR and sequencing results, Ta Khmau and Kandal cohorts, 2015. Adapted from Duong *et al*. [31].
(PDF)

S2 Table Detailed results of mosquito captures, July-October 2015, per site, night, and sex. A. July, B. August, C. September, and D. October.
(PDF)

S3 Table Comparison of ELISA and plaque reduction neutralization (PRNT) test results from 37 pig serum samples collected in 2006–2007. (PDF)
