# Supplementary material for: Comparison of the dynamics of Japanese encephalitis virus circulation in sentinel pigs between a rural and a peri-urban setting in Cambodia
Source: PLoS Negl Trop Dis. 2018 Aug 23;12(8):e0006644. doi: 10.1371/journal.pntd.0006644 (PMC6107123; doi:10.1371/journal.pntd.0006644)
Supplement: S1 File — (PDF) [file pntd.0006644.s001.pdf]

Household ID: ______ ___

Province: Kandal Ta Khmau

GPS coordinates:

How many people live in your household?
Children (≤ 15 years): ____
Adults (> 15 years): ____

Animal	Number of adults (>1 year)	Number of youngs (<1 year)
**Dog**		
**Cat**		
**Pig**		
**Chicken**		
**Duck**		
**Cattle**		
**Other**		

If you possess pigs, what type of farm do you have?
☐ Farrowing or ☐ Fattening or ☐ Farrow-to-finish or ☐ Backyard

Combien de portées de n porcs en moyenne naissent ou sont engraissées par an?
____ litters of ___ pigs born per year ; ____ litters of ___ pigs fattened per year

If you breed chickens, how many chicks are born every month?
____ chicks per month

Did you breed pigs last year? If yes, how many?
_____ Adults _____ Young
