# Supplementary figures and images for: Comparison of the dynamics of Japanese encephalitis virus circulation in sentinel pigs between a rural and a peri-urban setting in Cambodia
Source: PLoS Negl Trop Dis. 2018 Aug 23;12(8):e0006644. doi: 10.1371/journal.pntd.0006644 (PMC6107123; doi:10.1371/journal.pntd.0006644)

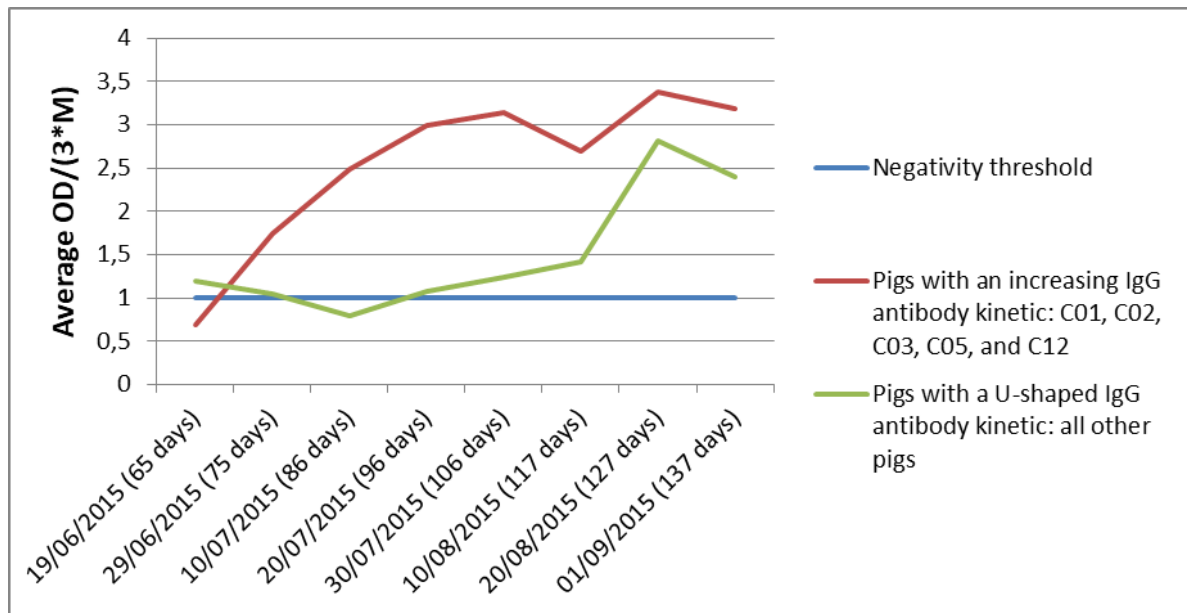

Supplement: S1 Fig — The optic density of each sample was divided by 3 times the mean absorbance of the three negative controls in the corresponding ELISA plate and then their average was calculated for each sampling date and type of IgG kinetic. The blue line corresponds to the negativity threshold, the red curve to the pigs with an increasing IgG antibody kinetic (C01, C02, C03, C05, and C12), and the green curve to the pigs with a U-shaped IgG antibody kinetic (all other pigs). (PDF) [file pntd.0006644.s002.pdf]

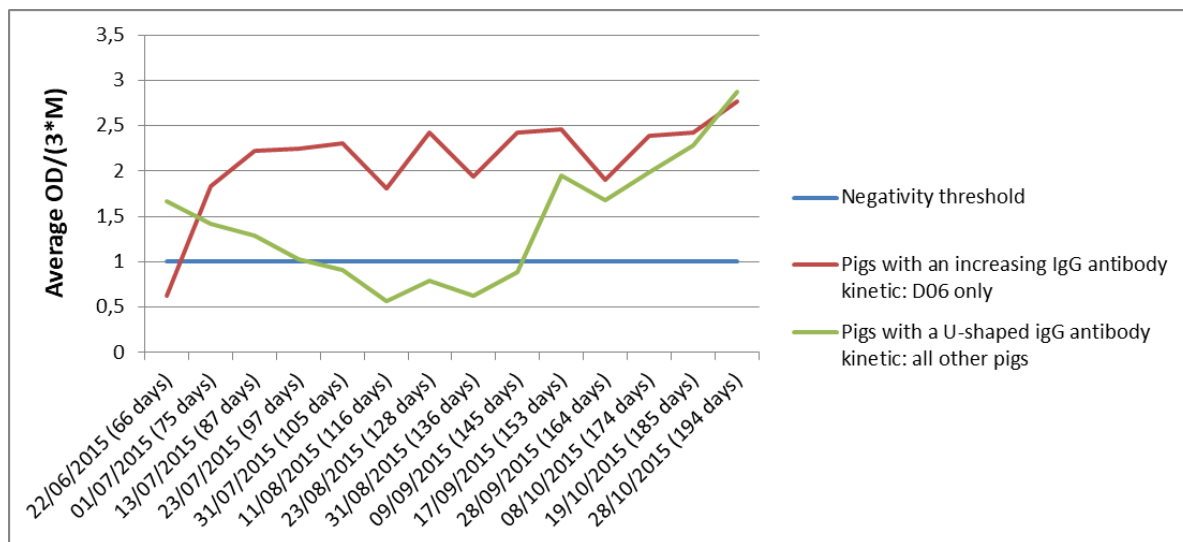

Supplement: S2 Fig — The optic density of each sample was divided by 3 times the mean absorbance of the three negative controls in the corresponding ELISA plate and then their average was calculated for each sampling date and type of IgG kinetic. The blue line corresponds to the negativity threshold, the red curve to the pigs with an increasing IgG antibody kinetic (D06 only), and the green curve to the pigs with a U-shaped IgG antibody kinetic (all other pigs). (PDF) [file pntd.0006644.s003.pdf]
