# Supplementary material for: Comparison of the dynamics of Japanese encephalitis virus circulation in sentinel pigs between a rural and a peri-urban setting in Cambodia
Source: PLoS Negl Trop Dis. 2018 Aug 23;12(8):e0006644. doi: 10.1371/journal.pntd.0006644 (PMC6107123; doi:10.1371/journal.pntd.0006644)
Supplement: S1 Table — Adapted from Duong et al. [31]. (PDF) [file pntd.0006644.s004.pdf]

**Piglet ID**	**Age (days)**	**Sex**	**Farm**	**Sampling Date**	**Real Time RT-PCR *(Ct value)***	**Conventional RT-PCR (NS3)**	**Virus isolation**	**Sequence (GenBank accession no)**	**Genotype**
C04	117	Male	Ta Khmau	10-Aug-15	Pos *(33.70)*	Pos	Neg	Partial NS3 (KY927813)	GI-b
C12	65	Male	Ta Khmau	19-Jun-15	Pos *(33.84)*	Pos	Neg	Partial NS3 (KY927810)	GI-b
C14	86	Male	Ta Khmau	10-Jul-15	Pos *(33.39)*	Pos	Pos	Full genome (KY927817)	GI-b
D03	145	Female	Kandal	09-Sep-15	Pos *(29.60)*	Pos	Pos	Full genome (KY927818)	GI-b
D08	145	Female	Kandal	09-Sep-15	Pos *(29.53)*	Pos	Neg	Partial NS3 (KY927811) Envelope (KY927814)	GI-b
D15	174	Male	Kandal	08-Oct-15	Pos *(29.26)*	Pos	Neg	Partial NS3 (KY927812)	GI-b
