# Supplementary material for: Comparison of the dynamics of Japanese encephalitis virus circulation in sentinel pigs between a rural and a peri-urban setting in Cambodia
Source: PLoS Negl Trop Dis. 2018 Aug 23;12(8):e0006644. doi: 10.1371/journal.pntd.0006644 (PMC6107123; doi:10.1371/journal.pntd.0006644)
Supplement: S2 Table — A. July, B. August, C. September, and D. October. (PDF) [file pntd.0006644.s005.pdf]

A. July

	Peri-urban							Rural						
	Night 1		Night 2		Night 3		Tot.	Night 1		Night 2		Night 3		Tot.
	♂	♀	♂	♀	♂	♀		♂	♀	♂	♀	♂	♀	
Culex tritaeniorhynchus	0	8	1	80	0	45	134	0	14	2	20	0	19	55
Culex gelidus	0	5	3	42	2	44	96	5	20	3	33	3	26	90
Culex vishnui	2	5	1	35	0	14	57	1	4	0	17	0	25	47
Culex quinquefasciatus	0	2	5	2	3	2	14	0	0	3	5	0	20	28
Culex sp.	0	0	0	0	0	0	0	0	0	0	0	0	0	0
Other	2	0	1	0	0	0	3	0	1	0	1	0	2	4
Total	4	20	11	159	5	105	304	6	39	8	76	3	92	224

B. August

	Peri-urban							Rural						
	Night 1		Night 2		Night 3		Tot.	Night 1		Night 2		Night 3		Tot.
	♂	♀	♂	♀	♂	♀		♂	♀	♂	♀	♂	♀	
Culex tritaeniorhynchus	9	284	6	323	5	220	847	0	27	0	120	2	159	308
Culex gelidus	12	372	23	510	10	256	1183	9	214	7	270	18	280	798
Culex vishnui	15	395	10	494	8	324	1246	2	52	0	72	4	181	311
Culex quinquefasciatus	15	1	3	0	7	6	32	10	6	0	0	4	1	21
Culex sp.	0	0	0	0	0	24	24	0	0	0	0	4	735	739
Other	0	7	3	16	0	2	28	1	3	0	3	0	11	18
Total	51	1059	45	1343	30	832	3360	22	302	7	465	32	1367	2195

C. September

	Peri-urban							Rural						
	Night 1		Night 2		Night 3		Tot.	Night 1		Night 2		Night 3		Tot.
	♂	♀	♂	♀	♂	♀		♂	♀	♂	♀	♂	♀	
Culex tritaeniorhynchus	5	363	1	216	0	161	746	5	290	4	64	1	58	422
Culex gelidus	74	489	33	669	26	362	1653	50	409	11	350	25	318	1163
Culex vishnui	18	393	4	128	9	191	743	4	267	7	106	0	117	501
Culex quinquefasciatus	1	0	0	0	4	0	5	21	7	4	8	0	3	43
Culex sp.	0	0	11	349	0	2	362	0	0	16	270	7	84	377
Other	0	4	0	3	0	11	18	0	31	0	25	0	16	72
Total	98	1249	49	1365	39	727	3527	80	1004	42	823	33	596	2578

D. October

	Peri-urban							Rural						
	Night 1		Night 2		Night 3		Tot.	Night 1		Night 2		Night 3		Tot.
	♂	♀	♂	♀	♂	♀		♂	♀	♂	♀	♂	♀	
Culex tritaeniorhynchus	7	361	11	735	5	858	1977	0	67	0	24	0	52	143
Culex gelidus	32	379	45	1072	42	1508	3078	2	92	0	62	2	126	284
Culex vishnui	13	557	12	880	0	690	2152	0	186	0	26	0	117	329
Culex quinquefasciatus	0	0	0	0	0	0	0	0	0	0	0	0	0	0
Culex sp.	0	0	19	278	0	1312	1609	0	26	0	302	0	230	558
Other	1	6	7	53	6	58	131	0	49	0	37	0	24	110
Total	53	1303	94	3018	53	4426	8947	2	420	0	451	2	549	1424
