# Supplementary material for: Comparison of the dynamics of Japanese encephalitis virus circulation in sentinel pigs between a rural and a peri-urban setting in Cambodia
Source: PLoS Negl Trop Dis. 2018 Aug 23;12(8):e0006644. doi: 10.1371/journal.pntd.0006644 (PMC6107123; doi:10.1371/journal.pntd.0006644)
Supplement: S3 Table — (PDF) [file pntd.0006644.s006.pdf]

Serum ID	Date of Sampling	Province of Origin	IgG ELISA	ELISA DO	Result PRNT 50 JEV	
C150	14.02.06	Kampong Cham	Neg	0.056	0	Neg
C142	03.02.06	Kampong Cham	Neg	0.015	0	Neg
C9	02.02.06	Kampong Cham	Neg	0.025	0	Neg
C151	14.02.06	Kampong Cham	Neg	0.066	0	Neg
C371	20.02.06	Kampong Cham	Neg	0.003	0	Neg
C116	03.02.06	Kampong Cham	Neg	NA	0	Neg
C24	02.02.06	Kampong Cham	Neg	0.042	0	Neg
C413	21.02.06	Kampong Cham	Neg	0.065	0	Neg
C1214062	14.12.07	Takeo	Neg	0.006	0	Neg
C1219091	19.12.07	Pursat	Neg	0.024	10	Neg
C305	20.02.06	Kampong Cham	Neg	0.068	15	Neg
C435	27.02.06	Kampong Cham	**Pos**	0.13	0	Neg
C205	14.02.06	Kampong Cham	**Pos**	0.149	0	Neg
C39	24.07.06	Kampong Speu	**Pos**	0.117	0	Neg
C363	20.02.06	Kampong Cham	**Pos**	0.208	0	Neg
C1205027	05.12.07	Kandal	**Pos**	0.229	147	**Pos**
C163	14.02.06	Kampong Cham	**Pos**	0.211	0	Neg
C168	14.02.06	Kampong Cham	**Pos**	0.313	0	Neg
C1214067	14.12.07	Kandal	**Pos**	0.406	69.7	**Pos**
C35	24.07.06	Kampong Speu	**Pos**	0.135	36	**Pos**
C140	03.02.06	Kampong Cham	**Pos**	0.325	98	**Pos**
C1212050	12.12.07	Kampot	**Pos**	0.203	253	**Pos**
C1214067	14.12.07	Kandal	**Pos**	0.406	69.7	**Pos**
C1205021	05.12.07	Svay Rieng	**Pos**	0.268	181	**Pos**
C1205024	05.12.07	Kandal	**Pos**	0.326	67	**Pos**
C1212056	12.12.07	Svay Rieng	**Pos**	0.375	102	**Pos**
C179	14.02.06	Kampong Cham	**Pos**	0.304	96	**Pos**
C183	14.02.06	Kampong Cham	**Pos**	0.189	328	**Pos**
C216	14.02.06	Kampong Cham	**Pos**	0.188	559	**Pos**
C1218079	18.12.07	Kandal	**Pos**	0.365	331	**Pos**
C1220098	20.12.07	Pursat	**Pos**	0.303	253	**Pos**
C1213057	13.12.07	Kandal	**Pos**	0.377	287	**Pos**
C461	27.02.06	Kampong Cham	**Pos**	0.372	200	**Pos**
C1206037	06.12.07	Takeo	**Pos**	0.226	496	**Pos**
C1207038	07.12.07	Takeo	**Pos**	0.357	785	**Pos**
C477	27.02.06	Kampong Cham	**Pos**	0.26	426	**Pos**
C1218072	18.12.07	Pursat	**Pos**	0.333	255	**Pos**
